# Blending Linear and Cyclic Block Copolymers to Manipulate Nanolithographic Feature Dimensions

**DOI:** 10.1021/acsapm.1c01313

**Published:** 2021-12-15

**Authors:** Amy D. Goodson, Maxwell S. Rick, Jessie E. Troxler, Henry S. Ashbaugh, Julie N. L. Albert

**Affiliations:** Department of Chemical and Biomolecular Engineering, Tulane University, New Orleans, Louisiana 70118, United States

**Keywords:** nanolithography, block copolymers, cyclic polymers, domain spacing, interfacial roughness, dissipative particle dynamics

## Abstract

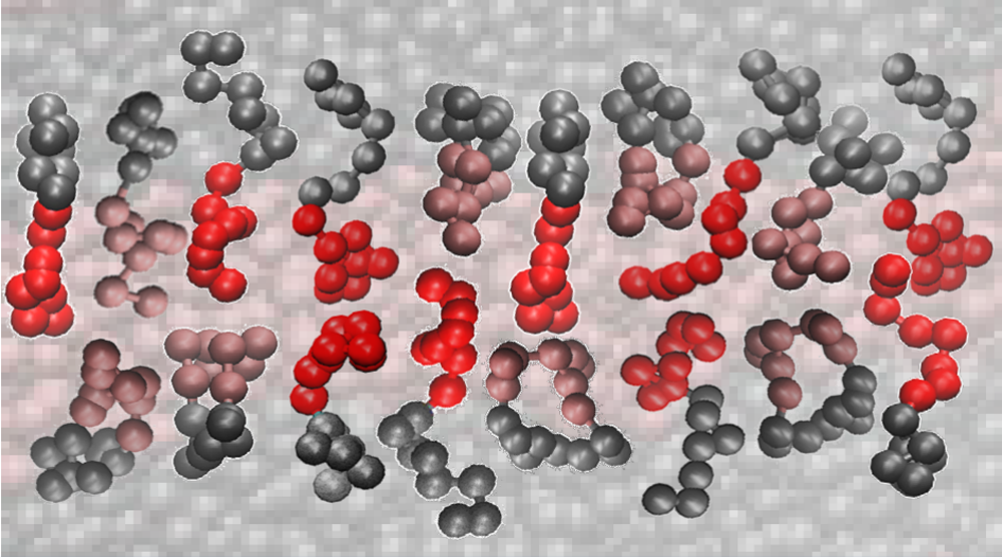

Block copolymers (BCPs) consist of two or more covalently bound chemically distinct homopolymer blocks. These macromolecules have emerging applications in photonics, membrane separations, and nanolithography stemming from their self-assembly into regular nanoscale structures. Theory suggests that cyclic BCPs should form features up to 40% smaller than their linear analogs while also exhibiting superior thin-film stability and assembly dynamics. However, the complex syntheses required to produce cyclic polymers mean that a need for pure cyclic BCPs would present a challenge to large-scale manufacturing. Here, we employ dissipative particle dynamics simulations to probe the self-assembly behavior of cyclic/linear BCP blends, focusing on nanofeature size and interfacial width as these qualities are critical to nanopatterning applications. We find that for mixtures of symmetric cyclic and linear polymers with equivalent lengths, up to 10% synthetic impurity has a minimal impact on cyclic BCP feature dimensions and interfacial roughness. On the other hand, blending with cyclic BCPs provides a route to “fine-tune” linear BCP feature sizes. We analyze simulated blend domain spacings within the context of strong segregation theory and find significant deviations between simulation and theory that arise from molecular-level packing motifs not included in theory. These insights into blend self-assembly will assist experimentalists in rationally designing BCP materials for advanced nanolithography applications.

## 1 Introduction

The exponential growth in computing power over the past fifty years can be largely attributed to advancements in photolithography, allowing for the number of transistors contained in a single silicon chip to double roughly every 18 months.^1,2^ However, as the microelectronics industry continues to demand smaller features, the cost of patterning devices by “top-down” methods increases exponentially.^1^ The 2017 Lithography Roadmap, which anticipates patterning technology requirements based on semiconductor customer needs, forecasts that high-performance logic devices will require sub-10 nm feature pitch by 2024.^3^ Realizing features this small by photolithography requires high-power, extreme ultraviolet light sources that are extremely expensive, and the resultant patterns often suffer from high defectivity.^1−3^ Nanopatterning by block copolymers (BCPs), which form nanoscale assemblies with dimensions dictated by polymer molecular weights, chemistry, and architecture, offers an attractive, lower cost method for production of next-generation logic and storage devices.^4,5^

BCPs consist of two or more covalently bound chemically distinct homopolymer blocks. Thermodynamic incompatibility between blocks drives phase separation; however, the covalent linkages limit segregation to the length scale of an individual polymer chain. As a result, BCPs self-assemble into a variety of nanoscale morphologies that minimize the free energy under the constraints imposed by the polymer’s composition and topology. Significant research has focused on the use of lamellar and cylindrical BCP morphologies, generally in combination with physical (graphoepitaxy) and chemical (chemoepitaxy) templates to improve long-range order, reduce defectivity, and allow for patterning of complex features, for next-generation microelectronics manufacturing.^5−7^ The ability to reliably pattern large-scale, defect-free, sub-10 nm features would significantly advance BCP nanolithography, meeting the microelectronics industry’s need for improved pattern resolution.

Ordered BCP nanostructures form only when the segregation strength (χ*N*, the product of the Flory–Huggins interaction parameter, χ, and the BCP degree of polymerization, *N*) is sufficiently high. In strongly segregated systems well above the order–disorder transition (ODT), i.e., χ*N* ≫ χ*N*|^ODT^, and in the absence of conformational asymmetry, nanostructure morphology is dictated by the volume fractions (*f*) of the different blocks. Meanwhile, feature size depends most strongly on *N*, which controls the overall polymer size, and weakly on χ, which characterizes the repulsion between the chemically distinct blocks. High χ systems allow for shorter chains (smaller *N* and therefore smaller feature sizes) to maintain order, but the large surface energy difference between blocks introduces additional challenges for BCP synthesis and assembly.^8−11^ BCP architecture provides an additional way to optimize nanofeature size.

Cyclic BCPs form domains significantly smaller than their linear analogues^12^ while exhibiting superior thin-film stability^13,14^ and assembly dynamics.^15,16^ Theory,^17,18^ simulation predictions,^19−21^ and experimental observations^12,22−24^ have all found that the phase diagram of cyclic BCPs resembles the linear phase diagram with the ODT shifted to a slightly higher segregation strength (χ*N*|_cyc_^ODT^ ≈ 1.7χ*N*|_lin_^ODT^) but with smaller nanostructures. Despite these desirable properties, the self-assembly of cyclic BCPs has not been investigated in detail due to challenges associated with achieving cyclic BCPs in sufficient quantity and purity for phenomenological study. While many of these challenges have been overcome through advancements in ring-closure chemistries and purification techniques,^25^ the need for pure cyclic BCPs would present a significant barrier to large-scale nanomanufacturing. Thus, we aspire to understand the self-assembly behaviors of cyclic and linear BCP blends with a focus on identifying the impact of synthetic impurities on the cyclic morphology as well as evaluating the potential for minimizing the use of the cyclic BCP by using it as an “additive” to direct the assembly of the linear polymer.

A multitude of different blends can be created by varying χ, *N*, *f_i_* (the fraction of *i* monomers in a polymer), and blend composition, described by the volume fraction of the cyclic polymer in the mixture, φ_cyc_. Molecular simulation allows for rapid exploration of these combinations to identify compositions that produce the desired nanofeatures, thereby shrinking the parameter space that must be examined experimentally. Herein, we use dissipative particle dynamics (DPD) simulations to investigate nanofeature sizes and interfacial widths formed by blends of cyclic and linear diblock copolymers (*i.e*., *AB* BCPs where *A* and *B* refer to the two blocks) of equivalent *N*. We investigate the impact of linear diblock and homopolymer impurities on symmetric (*f_A_* = *f_B_* = 0.5) cyclic BCP feature dimensions and discuss how adding cyclic BCPs could be used to fine-tune linear BCP feature sizes. We also analyze our simulated domain spacings in the context of strong segregation theory (SST) extended to BCP blends. Although we do not simulate thin-film confinement effects in this study, the insights provided by our simulations of blend self-assembly offer an initial guide to experimentalists for rationally designing BCP materials for advanced nanolithography applications.

## 2 Materials and Methods

### 2.1 Dissipative Particle Dynamics Simulations

DPD is a coarse-grained simulation technique that represents the BCP as a chain of soft beads, each representing tens of monomers, connected by Hookean springs, and interacting through soft pairwise forces. The high degree of coarse graining and softness of the interactions permit DPD to examine the phase behavior and mesoscale structure of polymer blends as functions of χ, *N*, *f_A_*, and chain architecture. Numerous simulations of BCPs with different topologies, such as cyclic, star, and π-shaped, have demonstrated that DPD captures the impacts of chain architecture on the polymer nanostructure including shifts in bulk morphology and feature size,^26−28^ solution micelle formation,^29−32^ and thin-film orientation.^33^ The Supporting Information contains a full description of the DPD model with references, but briefly, interparticle forces in DPD are broken up into a sum of pairwise conservative (**F**_*ij*_^C^), dissipative (**F**_*ij*_^D^), and random (**F**_*ij*_^R^) forces between particles *i* and *j*. Interactions between bonded particles are modeled using a Hookean spring (**F**_*ij*_^S^) that enforces bead connectivity and polymer architecture. We note that since DPD is a coarse-grained simulation technique (*N*_expt_ > 10*N*_DPD_), we cannot directly compare simulation and experimental values of χ. Rather, DPD values of χ are typically calculated to match experimental segregation strengths, i.e., χ*N*|_DPD_ = χ*N*|_expt_, to affect a meaningful comparison between simulation and experiment. For the remainder of the manuscript, *N* and χ refer to their values in a DPD simulation.

Although it is well established that non-concatenation requirements force cyclic homopolymers in a melt to be significantly more compact than Gaussian prediction,^34−38^ the soft potentials used in DPD may allow non-physical bond crossing to occur. However, the conformational impacts of non-concatenation increase with chain length, and very short cyclic molecules show Gaussian scaling even in the melt state.^34−36,39^ The high degree of coarse-graining in DPD makes DPD cyclic polymers necessarily very short, suggesting that they are in a regime in which bond crossing does not impact the conformational statistics. In addition, Huang *et al*. demonstrated that adding a spring–spring repulsion to the DPD model (thus preventing bond crossing) had a negligible impact on the morphologies and characteristic sizes of nanostructures formed by BCPs of several different topologies.^26^ Our previous simulation results for pure cyclic BCP domain spacing show good agreement with the experiment and strong segregation theory,^40^ suggesting that bond crossing has a negligible effect on the minimum energy feature morphologies and sizes formed in BCP self-assembly.

DPD simulations of symmetric linear (lin-*A_n_B_n_*) and cyclic (cyc-*A_n_B_n_*) BCP blends were performed using the LAMMPS software package.^41^ The subscript *n* indicates the number of monomers in each block so that the overall degree of polymerization is determined as *N* = 2*n* (*f*_*A*_ = *n*/2*n* = 0.5). All simulations utilized a value of χ = 12.2. Initially, cyc-*A_n_B_n_* was added to lin-*A_n_B_n_* in 10% increments to form blends that ranged from 0 to 100% cyclic polymer by volume; later work simulated blends at smaller increments of cyc-*A_n_B_n_* in areas of the phase diagram where interesting transitions in feature size and roughness were observed. Later, the impact of homopolymer synthetic impurities was studied by simulating blends of cyc-*A_n_B_n_* with *A_n_* and *B_n_* linear homopolymers. All blends consisted of 81,000 total beads in a periodic, cubic simulation box with side length *L* = 30, corresponding to a bead number density of ρ = 3. Simulations were started from random initial configurations and equilibrated for at least 10^6^ time steps. Following equilibration, production simulations were conducted for 5 × 10^5^ time steps. Structural quantities were calculated from configurations generated during the production run by averaging over a minimum of 50 configurations evenly sampled over the entire production run. All simulations were performed on the Ashbaugh group Dell cluster.

### 2.2 Structural Analysis

**Figure 1:**
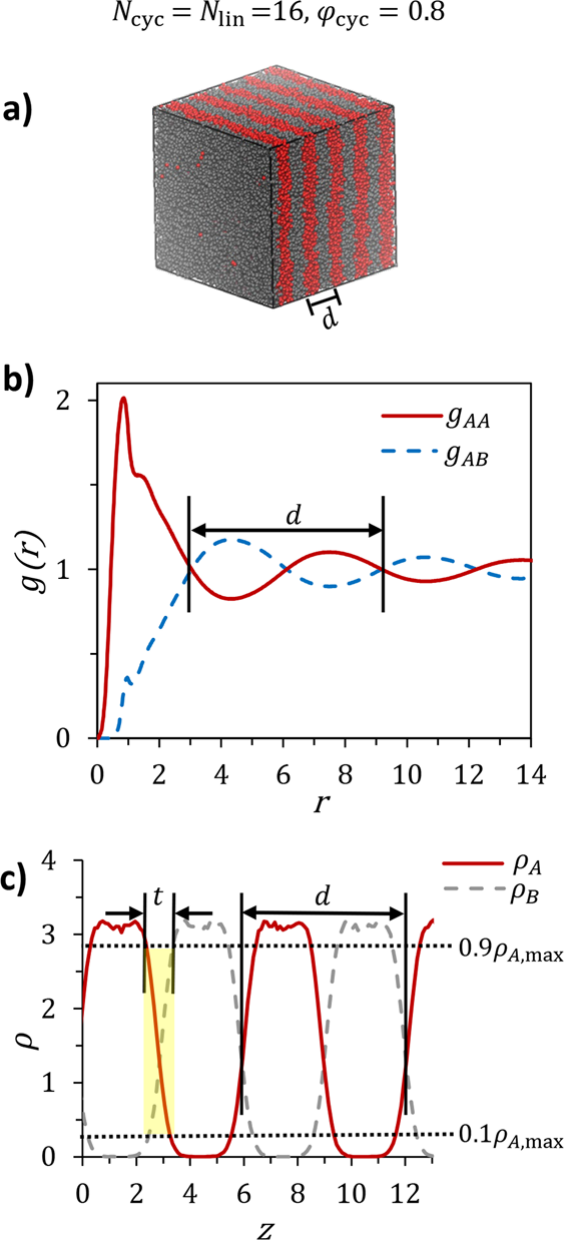
Methods for analyzing nanoscale features formed by a blend of symmetric (*f_A_* = 0.5) linear and cyclic BCPs with φ_cyc_ = 0.8: (a) VMD^43^ rendering of the equilibrated lamellar structure; (b) radial distribution functions, *g*(*r*), for the equilibrated lamellar structure. Domain spacing, *d*, is determined as the distance between the first and third (or *i* and *i* + 2) crossing points between *g_AA_*(*r*) and *g_AB_*(*r*). (c) Density profiles measured normal to the lamellar plane. Interfaces are represented by crossing points of ρ_*A*_(*z*) and ρ_*B*_(*z*), so *d* is the distance between the first and third (or *i* and *i* + 2) crossing points. Interfacial thickness, *t*, is defined as the distance normal to the lamellar interface over which  where ρ_*A*,max_ denotes the maximum measured value of *A* particle density in a given density profile.

Above the ODT, cyclic BCPs, linear BCPs, and their blends formed lamellae, consistent with experimental and theoretical phase diagrams (see for example, Figure 1a, a simulation snapshot of *N*_cyc_ = *N*_lin_ = 16, φ_cyc_ = 0.8). These lamellar structures were further analyzed using radial distribution functions (RDFs) and density profiles to measure domain spacing, interfacial roughness, and species segregation within lamellae.

The RDF, *g_ij_*(*r*), is a measure of the local density of particles of type *j* (*A* or *B*) a distance of *r* away from a reference bead of type *i* (*A* or *B*) normalized by the bulk density of *j*. As such, the RDFs provide critical information on the packing of particles of each type about another to provide insight into the three-dimensional structure system. The RDFs *g_AA_*(*r*) and *g_BB_*(*r*) subsequently provide information on the concentrations of like particles relative to each other, whereas *g_AB_*(*r*) (*g_AB_*(*r*) = *g_BA_*(*r*)) describes the correlations between unlike particles. In the symmetric lamellar morphology, the RDFs between like beads, *g_AA_*(*r*) and *g_BB_*(*r*), are equivalent and distinct from that between unlike beads, *g_AB_*(*r*). At low *r*, *g_AA_*(*r*) exhibits a peak and *g_AB_*(*r*) is suppressed, showing that on average, concentrations of *A* particles are the highest (and concentrations of *B* are the lowest) immediately next to other *A* particles, consistent with the segregation of particles into like domains. As we have previously demonstrated, the domain spacing (*d*) can be determined from the distance between the first and third crossing points between *g_AA_*(*r*) and *g_AB_*(*r*), which bound a single cycle of minima and maxima beyond the primary packing peak near *r* ≈ 1 as shown in Figure 1b.^42^

Concentration profiles for *A* and *B* monomers along the direction perpendicular to the lamellar interface provide another powerful visualization of the nanostructure (Figure 1c). In these profiles, interfaces are represented by crossing points of ρ_*A*_(*z*) and ρ_*B*_(*z*), so that *d* is determined from the distance between the first and third (or *i* and *i* + 2) crossing points. This density profile method provides an independent verification of the RDF domain spacing analysis while also providing valuable insights into how the cyclic and linear polymers arrange themselves within the lamellae, further explored in the Results and Discussion section.

We find that the *g*(*r*) and ρ(*z*) methods give *d* values within 1% of each other, providing confidence in our results. Resultantly, we only report domain spacings determined following the RDF method given its computational simplicity. However, both our qualitative discussions of cyclic/linear packing and quantitative analysis of blend interfacial thickness (see Results and Discussion) were extracted from the density profiles. Specifically, we calculated interfacial thickness, *t*, as the distance normal to the lamellar interface over which  as shown in Figure 1c. In this calculation, ρ_*A*,max_ represents the highest measured density of *A* particles in the profile being analyzed.

## 3 Results and Discussion

### 3.1 Domain Spacing

**Figure 2:**
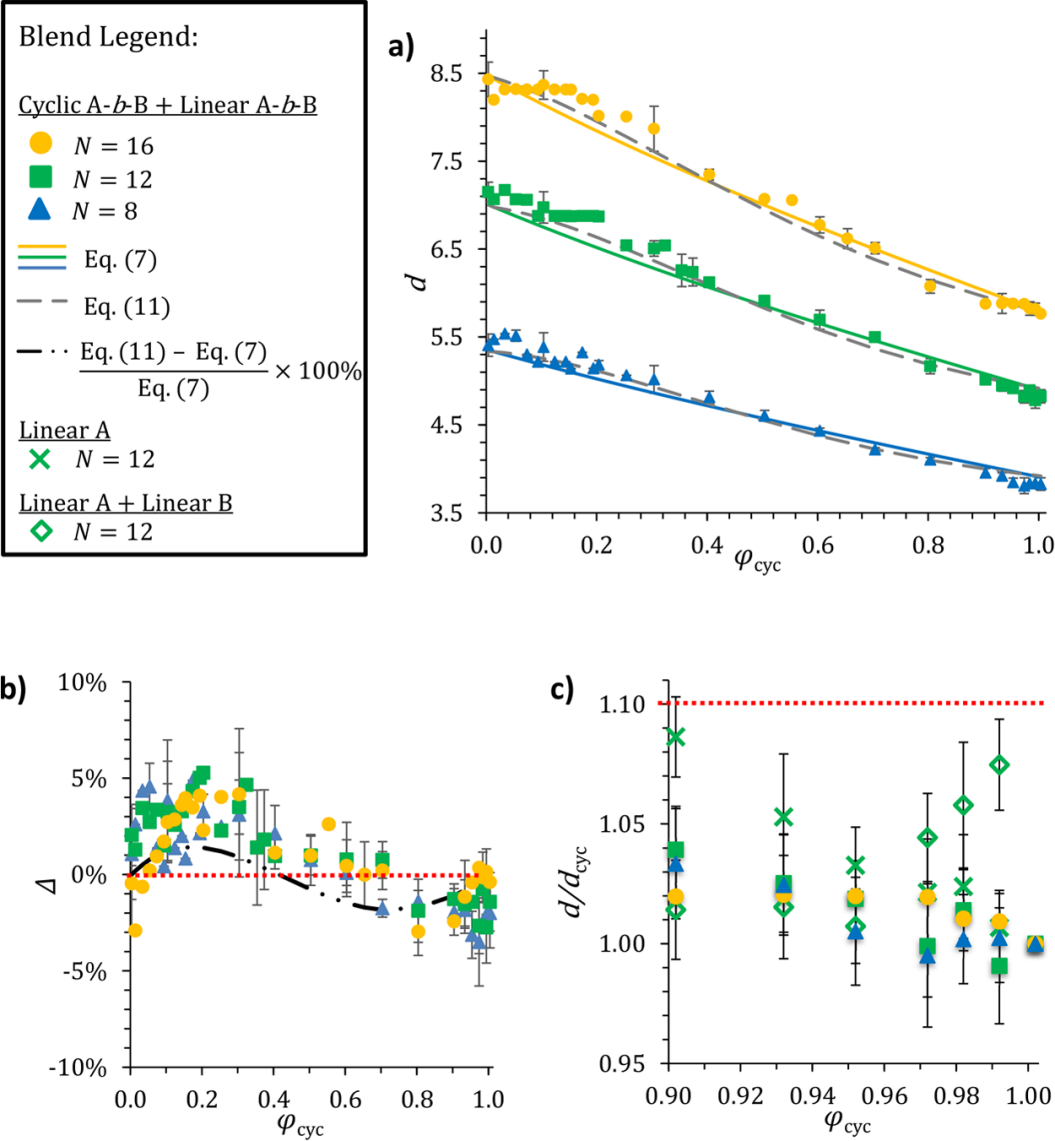
Effect of blending cyclic BCPs on nanoscale feature sizes formed by symmetric (*f_A_* = 0.5) linear BCPs. Data points and error bars represent the average and standard deviation, respectively, of *d* measured across three independent simulations. In several cases, the error bars are smaller than the data points. (a) Domain spacings of cyclic/linear BCP blends measured in DPD simulation (points) to those predicted by eq 7 (solid colored lines) and eq 11 (gray dashed lines). See text for equation details. (b) “Excess” domain spacing, Δ, defined as the percent difference between *d* measured from our DPD simulations (*d*_DPD_) and the eq 7 predictions (*d*_SST_) for a given cyclic/linear blend difference, i.e., . The dotted red line indicates perfect agreement between the simulation and eq 7, and the dot-dash line gives the Δ between eqs 7 and 11. (c) Impact of linear BCP and homopolymer impurities on cyclic BCP domain spacing. In linear *A* + linear *B* blends, some of the BCPs were replaced by equal volumes of *A* and *B* homopolymers, maintaining the overall composition. However, in linear *A* blends, some of the BCPs were replaced with *A* homopolymers resulting in a composition change from 50% *A* up to 55% *A* for the φ_cyc_ = 0.9 (10% impurity) blend. The dotted red line indicates a 10% increase in domain spacing relative to the pure cyclic BCP.

Figure 2a shows the domain spacings of cyclic and linear BCP blends as a function of φ_cyc_ at *N*= 8, 12, and 16 (corresponding to χ*N*= 98, 147, and 196, respectively; thus, all blend components exhibit SST scaling behavior as established previously by Goodson *et al*.^40^). In all three blends, the cyclic and linear polymers have the same number of monomers, so previous experimental^12^ and simulation^19,40^ work suggest that, for the pure components, *d*_cyc_ should be approximately 30% smaller than *d*_lin_ (*d*_lin_ ≈  for ideal random walk scaling). Indeed, we see that *d*(φ_cyc_ = 1)/*d*(φ_cyc_ = 0) (the ratio of the pure cyclic to pure linear BCP lamellar spacings, *d*_cyc_/*d*_lin_) ranges from 0.68 (*N* = 16) to 0.71 (*N* = 8). To a first approximation, the blend feature size linearly decreases from the pure linear to pure cyclic domain spacings as a function of the blend composition. However, notable deviations are observed, as discussed in the following paragraphs.

Because the values of χ*N* in these simulations are well above the order–disorder transition and in a regime where SST scaling behavior has been established previously,^40^ we can rationalize our simulation observations by extending SST^44−46^ to polymer blends to describe the dependence of the domain spacing on blend composition. Our application of SST principles differs from prior SST- and mean-field theory work related to linear–linear BCP blends^47−49^ because our framework also draws on prior work related to the scaling behavior of cyclic BCPs compared to their linear counterparts.^40^ In the original development of SST, the free energy of a pure BCP melt is expressed as a linear combination of contributions due to unlike monomer contact at the interface between domains, which favors domain swelling to minimize inter-block contact and chain stretching, which opposes domain growth that drives chains away from their ideal Gaussian confirmations.^45,50^ This free energy of a single chain in a pure BCP lamella can subsequently be expressed as
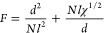
1where the first term in this expression represents the stretching penalty as a Hookean spring, the second represents the contact penalty that acts to minimize *A* and *B* interfacial growth, and *l* is the characteristic segment length of the polymer. The equilibrium domain spacing is obtained by minimizing *F* with respect to *d*, yielding
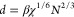
2where the prefactor β is .

Comparing the predictions of eq 2 against simulation results for pure linear and cyclic BCPs, we previously phenomenologically revised eq 2 to account for both polymer architecture and localized chain stretching at the domain interface.^40^ The resultant expression for the BCP domain sizes mirrors eq 2
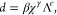
3where, β and χ assume the same role as in eq 2, but *N* is replaced by the polymer “extent” embodied by the parameter Λ. The exponents γ and ε are theoretically equal to 1/6 and 2/3, respectively, although they were left as adjustable parameters to obtain improved agreement with the simulation. Parameter Λ consists of an architecture-dependent term that grows with *N* modified by a correction to capture the fact that the bonds between unlike monomers seated at a lamellar interface, *b_AB_*, are ∼35% longer than those between like monomers, *b_AA_* = *b_BB_*.^40^ This approach gives the following definitions for cyclic and linear BCPs, respectively:
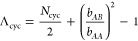
4a
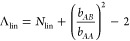
4bAs the polymer chains become longer, the extents asymptotically approach Λ_lin_ → *N*_lin_ and Λ_cyc_ → *N*_cyc_/2 as expected based on random walk scaling. For the short, coarse-grained polymers simulated here, however, the impacts of finite polymer length and localized stretching of the bonds at the interface (*b_AB_* > *b_AA_*) must be taken into account.

In fitting eq 3 to pure linear and cyclic BCP domain spacings, the ratio *b_AB_*/*b_AA_* was directly evaluated from the simulation, while β, γ, and ε were left as adjustable parameters. Excellent agreement between the simulation and theory was obtained from the values reported in Table 1. The fitted exponents γ (0.140) and ε (0.653) closely match those predicted by SST, providing confidence in the physics underlying the model.^40^ Incorporating these architecture-dependent contributions into the free energy of an individual chain of a pure BCP yields
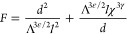
5This expression returns eq 3 when minimized with respect to *d*. This expression serves as the starting point for modeling blends.

**Table 1:** Fit Parameters Used for Eqs 6 and 9

β	0.832
γ	0.140
ε	0.653
*b_AB_*/*b_AA_*	1.356

Given that eq 5 accounts for the free energies of both linear and cyclic chains, the simplest extension of this expression to blends would be to assume additivity of the free energy of the individual components. The mean free energy of a chain in the blend then can be modeled as the sum of the free energies of the linear and cyclic chains multiplied by their respective mole fractions.
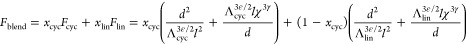
6where *x_i_* is the mole fraction of component *i* (lin or cyc) in the blend. Although the Flory–Huggins mixing entropy contributes to the overall free energy, it does not depend on the domain spacing. As a result, the mixing entropy will not impact the domain spacing obtained when *F*_blend_ is minimized with respect to *d*, so we neglect it here. Minimizing *F*_blend_ with respect *d* yields the blend scaling law
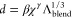
7awhere
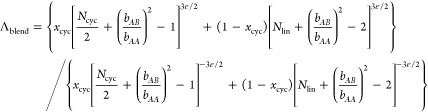
7bSince no additional fitted parameters were incorporated into the blend model, eq 7 can be applied directly using the parameters listed in Table 1.

The predictions of eq 7 are depicted by the solid lines in Figure 2a. In our simulated blends, the monomers occupy identical volumes and *N*_lin_ = *N*_cyc_ = *N*, so the mole fraction, *x*_cyc_, and volume fraction, φ_cyc_, are equivalent. For consistency with the experimental literature on polymer blends, our results are plotted and discussed in terms of φ_cyc_. While eq 7 semiquantitatively captures the overall simulation trends, there are clear discrepancies between the two approaches. In particular, eq 7 predicts a nearly linear decrease in the domain spacing as a function of φ_cyc_, while the simulations exhibit small positive/negative deviations from predictions at low/high cyclic blend compositions. To quantify these deviations, we calculate the “excess” domain spacing, Δ, defined as the percentage difference between *d* measured from our DPD simulations (*d*_DPD_) and the eq 7 predictions (*d*_SST_) for a given cyclic/linear blend:
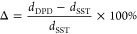
8The “excess” domain spacings for simulations at different degrees of polymerization are comparable to one another (Figure 2b), suggesting that these differences result from a mixing effect between the linear and cyclic chains unaccounted for by the theory. Clearly, the scaling theory underpredicts the blend domain spacing (Δ > 0) for 0 ≤ φ_cyc_ ≤ 0.4, with the discrepancy reaching its maximum (5%) near φ_cyc_ ≈ 0.3. Above φ_cyc_ = 0.8, on the other hand, simulation gives smaller domain spacings than would be predicted by eq 7 (Δ < 0), although the discrepancy is not as large as for the linear-rich blends. These deviations are further explored below.

### 3.2 Density Profiles

**Figure 3:**
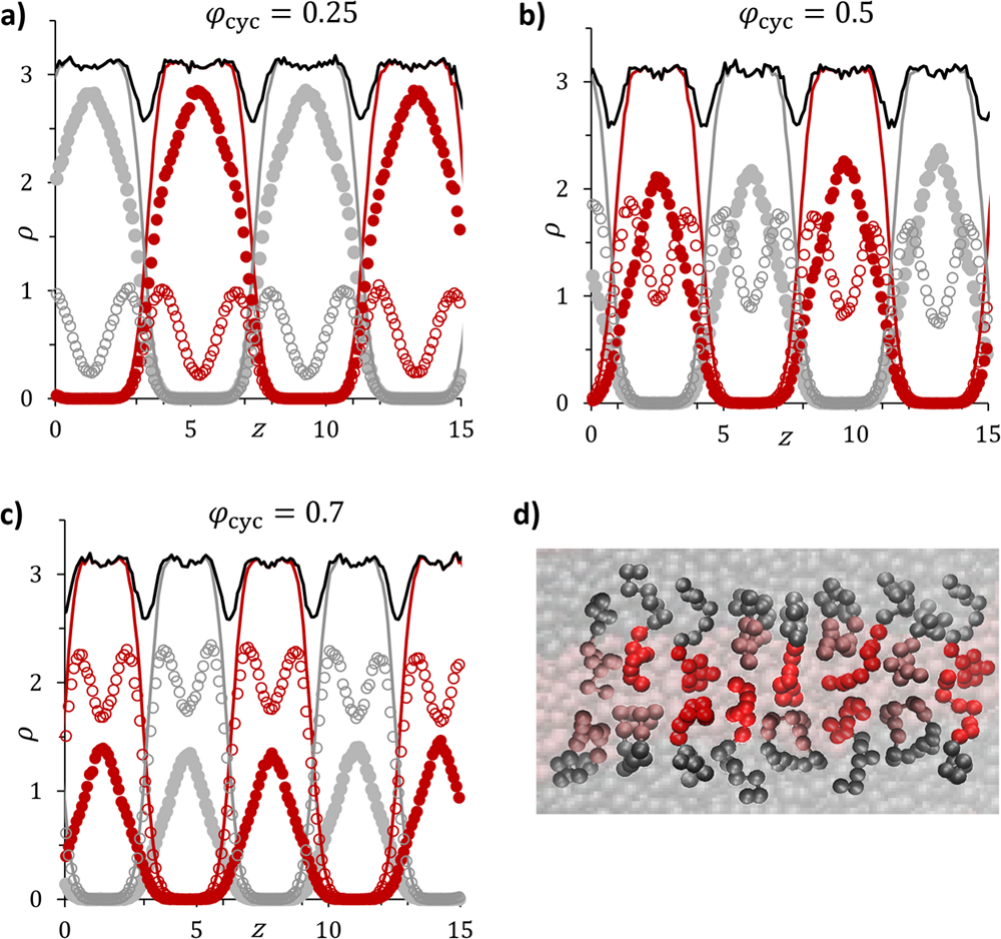
Concentrations of cyclic *A* (open red circles), cyclic *B* (open gray circles), linear *A* (solid red circles), and linear *B* (solid gray circles) along the direction normal to the lamellar interface in cyclic/linear BCP blends (*N* = 16) with (a) φ_cyc_ = 0.25, (b) φ_cyc_ = 0.5, and (c) φ_cyc_ = 0.7. In all three figures, the lines represent the total block A (red line), block B (gray line), and overall (black line) monomer concentrations. (d) Snapshot from the DPD simulation of a cyclic/linear BCP blend with φ_cyc_ = 0.3. Sample conformations of individual cyclic (light shading) and linear (brighter shading) chains have been highlighted to demonstrate the molecular packing described in the text.

To assess the distribution of linear and cyclic BCP monomers within blended lamellar domains as well as quantify nanofeature roughness, we calculate the density distributions along the direction normal to the lamellar interface. Figure 3a–c shows example blend density profiles at varying φ_cyc_ values, depicting the relative concentrations of four different types of monomers (cyclic *A*, cyclic *B*, linear *A*, and linear *B*) as well as the total *A*, total *B*, and overall blend densities along a path normal to the lamellar interface. All density traces display a regular periodicity, implying that the different architectures are well dispersed throughout the simulation box. This behavior is consistent with theoretical predictions that cyclic and linear homopolymers display enhanced miscibility.^34,39,51^ However, the cyclic and linear molecules clearly arrange themselves differently relative to the lamellar interface. Within an individual lamellar domain, the distribution profile of the cyclic molecules exhibits two peaks, one at each *A*–*B* interface. However, the linear BCPs show a single peak at the center of the *A* domain and at the center of the *B* domain, suggesting that, on average, linear molecules extend further into the domains than the cyclic molecules do, as illustrated in the simulation snapshot provided in Figure 3d. In contrast, eq 6 assumes that cyclic and linear polymers are equally stretched (i.e., share a single value for *d*). This discrepancy between the simulation results and the theoretical assumption is explored further below (see Molecular Conformations).

### 3.3 Interfacial Roughness

**Figure 4:**
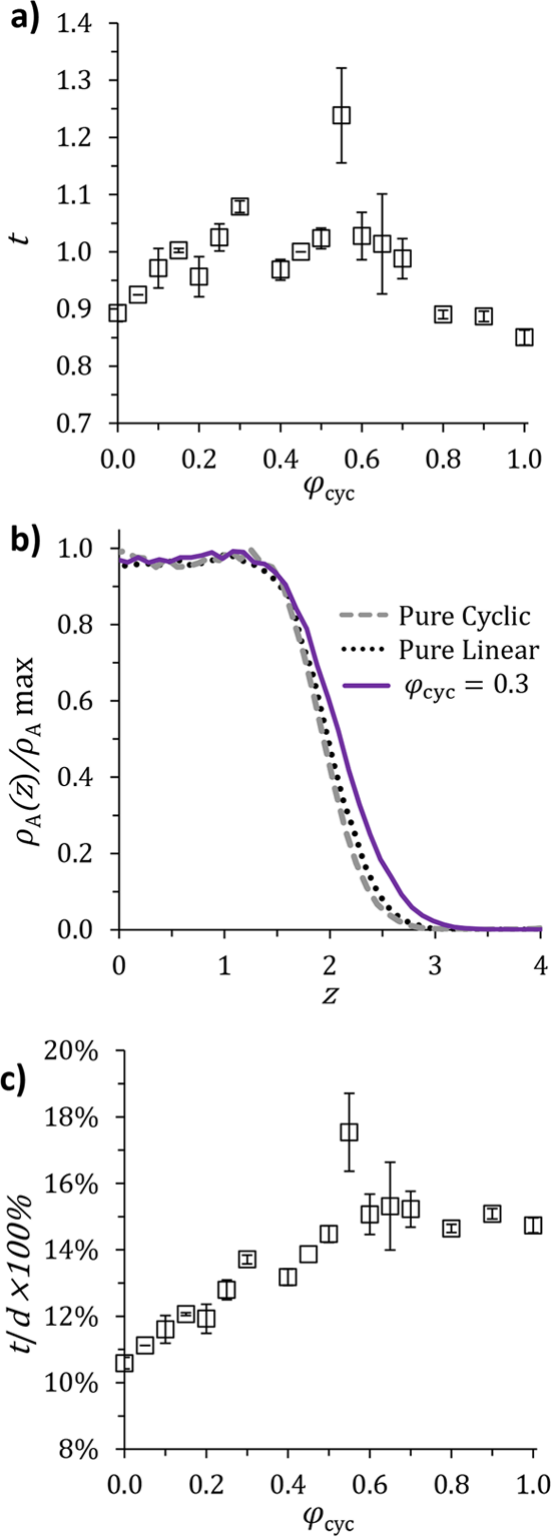
Interfacial widths of cyclic/linear BCP blends of *N* = 16. (a) Effect of blending on nanofeature interfacial thickness, *t*, defined as the distance normal to the lamellar interface over which  (see Figure 1c). (b) Illustration of an average interfacial concentration profile for a pure linear BCP (φ_cyc_ = 0, dotted black line), a pure cyclic BCP (φ_cyc_ = 1, dashed gray line), and a linear/cyclic BCP blend (φ_cyc_ = 0.3, solid purple line). The shaded area represents the interfacial region as defined above. (c) Interfacial thickness, *t*, normalized by domain spacing, *d*. This ratio represents the fraction of each lamellar nanofeature contained in the interface. In (a) and (c), error bars represent the standard deviation of *t* (or *t*/*d*) across three independent simulations of each blend.

We can use the total *A* and *B* density distribution profiles from the simulation to quantify the nanofeature interfacial width, a quality critical to successful nanolithography pattern transfer. We define the lamellar interface as the region in which *A* particle density is between 10 and 90% of ρ_*A*,max_ and quantify interfacial thickness, *t*, as the distance normal to the lamellar interface over which  (see Figure 1c). The thickness *t* includes impacts of both short and long wavelength fluctuations in the lamellar interface, which correlate with line-edge roughness and interfacial curvature, respectively. Figure 4a shows how this quantity varies with φ_cyc_ in blends of cyclic and linear BCPs with *N* = 16. Interfaces are the smallest for the pure components (φ_cyc_ = 0 and 1) and widen as the blends become more symmetric. Figure 4b provides another illustration of this trend, showing how the density of *A* monomers (normalized by ρ_*A*,max_) changes as one moves along the lamellar normal. Here, a sharper (larger magnitude) slope of ρ_A_(*z*)/ρ_*A*,max_ in the interfacial region represents a narrower interface. Clearly, the φ_cyc_ = 0.3 blend interfaces are wider than those formed by pure cyclic or linear polymers. Taken together, Figure 4a,b suggests that blending BCP architectures increases interfacial roughness of the nanofeatures formed.

To better understand how this trend would affect an experimental system, we normalize interfacial thickness by domain spacing to calculate the percentage of each lamellar period contained in the interfacial region. This percentage, (*t*/*d*) × 100 % , is plotted in Figure 4c. The pure linear BCP (φ_cyc_ = 0) has an interfacial width corresponding to 10% of its total lamellar spacing. As the cyclic BCP is added, *t*/*d* increases until it reached a plateau of ∼15% for φ_cyc_ ≳ 0.6. For a 10 nm lithographic feature, these values correspond to 1 nm of each lamellar leaf existing as a part of the interface in a pure linear BCP as compared to interfaces being 1.5 nm wide when φ_cyc_ > 0.6. Thus, even though the absolute values found for the interfacial roughness of pure linear BCPs and pure cyclic BCPs are similar, taken as a fraction of the domain spacing, the cyclic BCP has wider interfaces; these results are consistent with the experimental findings of Gartner *et al*.^24^

### 3.4 Applications to Nanolithography

The overall goal of this work is to investigate the potential of self-assembling cyclic BCPs as nanotemplates. We now explore the simulation results described above to quantify (1) the impact of linear impurities on cyclic BCP self-assembly and (2) the feasibility of using cyclic BCPs as a structure-directing agent. The simulations also provide insights into how cyclic and linear chain conformations change with blend composition and how these molecular-level properties impact nanoscale feature sizes.

Cyclic BCPs are most often produced by sequential synthesis and end-group functionalization of a linear *ABA* triblock copolymer followed by a single click reaction to create the ring structure. Another approach involves synthesis of two telechelic homopolymer blocks followed by two click reactions to create the interfacial bonds.^12^ Cyclic synthetic impurities, then, may include excess homopolymer, linear *AB* and *ABA* copolymers, and/or linear and cyclic multiblock products. Experimentally, high dilution minimizes multiblock production and the high efficiency of the click reaction means that end-functionalized linear precursors can be assumed to react completely.^12,52,53^ However, incomplete conversion of end groups on linear precursors (e.g., bromide to propargyl or azide for copper-catalyzed azide-alkyne click coupling) may result in the presence of linear homopolymer, diblock copolymer, and/or multiblock copolymer impurities that are difficult to detect and even more difficult to remove.^54−56^ For cyclic homopolymers, 1% linear impurity is known to cause dramatic changes in rheologic properties and diffusivity^39,57^ but has a minimal effect on molecular dimensions.^37^ Meanwhile, homopolymer impurities swell linear BCP domain spacings, increase interfacial fluctuations, and even accelerate self-assembly processes under some conditions.^58−61^ These varied results indicate that acceptable cyclic polymer purity will be highly dependent on the end application. A wealth of BCP processing techniques, including solvent vapor annealing, thermal zone annealing, and shear alignment,^62^ are available to overcome challenges in polymer processing and self-assembly dynamics, which may be introduced by blending linear and cyclic architectures, so we focus on the impact of impurities on equilibrium feature sizes and interfaces.

Figure 2c shows that cyclic domain spacing is insensitive to the presence of linear impurities with both simulation and scaling law results, indicating that features will swell less than 5% when a cyclic BCP contains up to 10% linear impurity (φ_cyc_ = 0.9). For a 10 nm experimental feature, impurity effects observed here would translate as a <0.5 nm domain spacing increase, putting the impacts on the same order of magnitude as those of experimental measurement precision. Homopolymer impurities have a slightly stronger impact on cyclic feature size, but the maximum homopolymer-induced domain spacing increase is still less than 10% for 10% impurity, regardless of whether the impurity consists entirely of one type of homopolymer or both *A* and *B* homopolymers. Also, up to 10% linear impurity has negligible impact on cyclic BCP interfacial width, as illustrated in Figure 4a,c. Although not simulated, we would expect that linear or cyclic multiblock impurities, whose constituent blocks would have the same molecular weight as the majority cyclic BCPs, would likewise have a minimal impact on the domain spacing in this regime, perhaps exhibiting behavior intermediate to that seen for linear diblock and linear homopolymer impurities. We conclude that up to 10% linear impurity has a minor impact on feature size and interfacial roughness, indicating that an extremely high-purity material is not required for use of cyclic BCPs in nanolithography applications. This finding suggests that costly post-synthesis purification can be avoided, providing greater freedom in optimizing reaction conditions to create more scalable cyclic BCP syntheses.

Conversely, the low φ_cyc_ side of Figure 2a shows that the cyclic BCP is not an effective structure-directing agent. Just as self-assembly of a pure cyclic BCP is relatively insensitive to small amounts of linear impurity, it appears that small concentrations of the cyclic BCP have a limited ability to shrink feature sizes of self-assembling linear molecules; more than 30% cyclic content would be required to shrink a 10 nm linear BCP feature by 1 nm.

Instead, blending the different polymer architectures may hold promise as a method for precisely tuning feature dimensions to meet advanced lithography demands.^12,61,63,64^ For example, defect-free epitaxial self-assembly requires a BCP that can form features commensurate with template pattern dimensions; irregular geometries such as bends are particularly sensitive to non-commensurability.^6,61,64^ BCP blending may also provide access to multiple structure dimensions and geometries on a single device. In employing this approach, experimentalists must consider the effect of blending on interfacial width, which increases from 10% of the lamellar width for the pure linear molecule to 15% in the φ_cyc_ = 0.5 blend (Figure 4b). The acceptable interfacial roughness will vary with the exact feature morphology and size being used in a particular patterning application. Taken together, Figures 2 and 4 quantify the trade-off between decreasing feature size and increasing interfacial width in cyclic/linear BCP blends, providing an initial roadmap for experimentalists combining molecular architectures to achieve a desired feature size.

### 3.5 Molecular Conformations

In addition to providing experimental insights, our simulations allow us to probe the conformations and packing motifs of cyclic and linear BCPs in blends and understand how their properties impact nanoscale self-assembly. The density profiles in Figure 3a–c indicate that, in all cyclic/linear BCP blends, the cyclic polymer concentration peaks at lamellar interfaces, whereas domain centers are enriched in the linear counterpart. These results can be explained by the microphase separation process that localizes bonds between *A* and *B* blocks at the *A*–*B* interface, with the centers-of-mass of the *A* and *B* blocks pointing toward their corresponding domain center. Because the effective size of a cyclic molecule is always less than that of its linear analog with the same degree of polymerization, the packing signatures that minimize *F*_blend_ involve linear polymers adopting a “dumbbell” shape in which the bulk of the block mass is segregated in the center of the domain, while the interfacial bond is at the interface, consistent with the density profiles shown in Figure 3. This linear polymer conformation then allows the cyclic polymers to maintain more compact configurations with the majority of cyclic monomers sitting in close proximity to the interface, as shown in the simulation snapshot provided in Figure 3d. These results are consistent with previous work on polydisperse linear BCPs, where lamellar interfaces were found to be enriched with shorter molecules, while the centers-of-mass of the longer molecules were concentrated at domain centers.^42,65−67^

**Figure 5:**
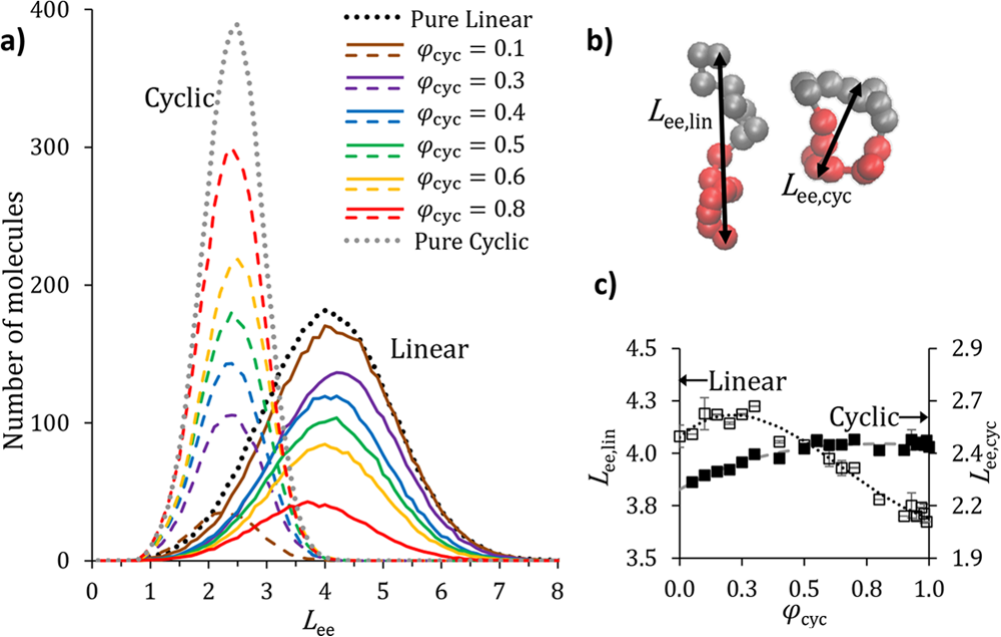
(a) End-to-end length, *L*_ee_, distributions of cyclic and linear polymer chains in different blends as defined in the legend. While these separation distributions could be normalized so that the integral under the curve is one, normalization obscures the trends observed with changing blend compositions. Thus, we chose not to normalize these distributions for clarity. (b) For linear molecules, *L*_ee_ is calculated as the total distance between the first type *A* and last type *B* particles. In cyclic molecules, the quantity is measured as the span between the center of the *A* and *B* blocks. (c) Mean *L*_ee_ for linear (*L*_ee, lin_; left axis, open points) and cyclic (*L*_ee, cyc_; right axis, solid points) BCPs calculated from a Gaussian fit of *L*_ee_ distributions. Error bars represent the range of values measured in two independent trials. The black dotted (linear) and gray dashed (cyclic) lines show the polynomial fits (*L*_ee_ = *a*φ_cyc_^3^ + *b*φ_cyc_^2^ + *c*φ_cyc_ + *d*) used to calculate δ^2^ as described in the text. For *L*_ee, lin_, *a* = 1.49, *b* = 2.93, *c* = 1.06, and *d* = 4.07. For *L*_ee, cyc_, *a* = 0.230, *b* = 0.705, *c* = 0.691, and *d* = 2.23.

While the linear BCP “dumbbell” motif is present in all blends studied (see Supporting Information for additional density profiles), linear and cyclic molecule conformations do appear to shift with blend composition. Figure 5a shows distributions of cyclic and linear molecule end-to-end lengths, *L*_ee_, in *N*_DPD_ = 16 blends. For linear polymers, *L*_ee_ is the distance between the first (1) and last (*N*) monomers. For cyclic polymers that have no ends, we defined *L*_ee_ as the distance between the midpoints of the *A* and *B* blocks (the midpoint of the *N*/4 and *N*/4 + 1 monomers and the 3*N*/4 and 3*N*/4 + 1 monomers, as counted from the first monomer in the *A* block). These distances for the linear and cyclic polymers are illustrated in Figure 5b. Figure 5c reports the mean *L*_ee_ value for each blend, which was extracted from fitting the curves in Figure 5a to a Gaussian model.

Starting on the left side of Figure 5c with the pure linear BCP (φ_cyc_ = 0), we can see that adding the cyclic BCP to the linear BCP weakly swells the linear molecule, even as the overall lamellar spacing slightly decreases. In the range of 0 ≤ φ_cyc_ ≤ 0.3, the linear molecule stretches beyond its pure component configuration (*L*_ee, lin_ = 1.04*L*_ee, φ = 0_, when φ_cyc_ = 0.3). For φ_cyc_ > 0.3, however, increasing cyclic content shrinks the linear BCPs. Meanwhile, the cyclic molecules are most compressed at small φ_cyc_ and continue to stretch until they reach the pure cyclic *L*_ee_ value at approximately φ_cyc_ ≈ 0.6. In the majority of cyclic blends, linear BCPs contract as φ_cyc_ increases and the overall domain spacing shrinks to accommodate the high concentration of cyclic molecules; the cyclic dimensions are relatively constant in these blends.

This non-linear behavior of molecular dimensions with φ_cyc_ manifests in nanoscale feature sizes. At a low cyclic content, adding cyclic molecules causes the linear BCP “dumbbell” to stretch into the domain center to minimize cyclic polymer stretching and avoid the associated free-energy penalty. More than 30% cyclic content is required to reverse this behavior and drive compression of the linear polymer configuration so that blend features shrink as predicted by eq 7. This weak response limits the potential of cyclic BCPs as structure-directing agents for linear molecules. A similar effect is visible on the high cyclic content side of the phase diagram (see Figure 2a,b) where eq 7 slightly overpredicts the simulation domain spacing for blends with 0.8 ≤ φ_cyc_ ≤ 1. Figure 5c shows that cyclic molecule end-to-end lengths are relatively constant in this blend composition range. Again, we see that approximately 30% of the minority component (linear BCP in this case) is required to drive dramatic changes in majority (cyclic) component dimensions, manifesting as the cyclic BCP is being relatively insensitive to synthetic impurities.

Finally, investigating molecular dimensions provides insights into the discrepancies between scaling law predictions and simulation results for blend domain spacing, as shown in Figure 2b. Specifically, Figure 5c makes it clear that blend molecules can be significantly stretched or compressed relative to their pure component conformations, and that these dimensions change with φ_cyc_. In these blend packing arrangements, then, the size of a molecule is not fully captured by Λ as defined in eq 4.

We can adjust Λ to account for how stretched or compressed a BCP is in a given blend by multiplying it by the squared ratio of *L*_ee_ for the blend molecule to the *L*_ee_ of the pure component:
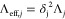
9where
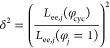
10In eq 10, *j* denotes the subscripts cyc and lin and *L*_ee_(φ_*j*_ = 1) is the pure component end-to-end length. Random walk statistics,^68−70^ which underlies the development of the revised scaling law (eq 3),^40^ predict that *L*_ee_ should scale as *N*^1/2^, i.e., *N* ∝ *L*_ee_^2^. In eqs 3–7, Λ takes the place of *N*, so it follows that Λ should be adjusted by the squared ratio of end-to-end lengths to capture the impact of chain stretching and compression.

Replacing Λ with Λ_eff_ yields a modified blend scaling law:
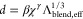
11awhere
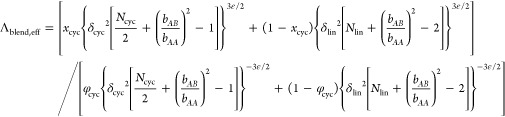
11bTo apply eq 11, we fit the *L*_ee, cyc_ and *L*_ee, lin_ values measured for blends with *N* = 16 as third degree polynomials in φ_cyc_ (see Figure 5c) and then use these fit equations to calculate δ^2^. Figure 2b suggests that the blend packing trends are independent of chain length, so we use these same δ^2^ values for the *N* = 8 and *N* = 12 blends to produce all of the dashed lines in Figure 2a. Comparing eq 11 to the simulated domain spacings gives a root-mean-square (RMS) error of 0.126, less than 3% of the smallest measured value of *d* in this work. For comparison, the RMS error in eq 7 is 0.170.

As another illustration of eq 11’s superiority in predicting *d*, we plot the “excess” domain spacing for eq 11 relative to eq 7 as a dot-dash line in Figure 2b. The close match between this line and the simulated values of Δ (data points) illustrates how eq 11 captures the system’s resistance to change from pure component domain spacings on both sides of the phase diagram. We therefore conclude that, while the original blend scaling law and even an assumption of linear scaling can provide a qualitative understanding of feature size in cyclic/linear BCP blends, accurate quantitative prediction of *d* requires an understanding of how linear and cyclic polymers pack in blends.

## 4 Conclusions

We have systematically investigated the self-assembly behavior of cyclic/linear BCP blends and explored these findings in the context of exploiting cyclic BCPs for tuning feature sizes in nanolithography. We find that up to 10% linear diblock or homopolymer impurity has a minimal impact on cyclic BCP feature size and interfacial width, suggesting that an extremely high-purity material is not required for use of cyclic BCPs in nanolithography applications. Similarly, adding small concentrations of cyclic BCPs has virtually no effect on linear self-assembled feature size or roughness, leading us to conclude that cyclic BCPs are not effective structure-directing agents. Instead, blending the different molecular architectures may hold promise as a method for precisely tuning feature dimensions to meet advanced lithography demands. We also find that blend packing arrangements cause both linear and cyclic chains to stretch or compress such that their effective size is distorted from strong segregation theory predictions. Therefore, accurate quantitative prediction of blend feature size requires an understanding of blend packing motifs. These insights into material purity requirements and chain packing will assist selection of BCP materials for nanolithography applications.

## Supporting Information Available

DPD simulation methods and density profiles calculated from blend simulations (PDF)
