# Supplementary material for: Blending Linear and Cyclic Block Copolymers to Manipulate Nanolithographic Feature Dimensions
Source: ACS Appl Polym Mater. 2021 Dec 15;4(1):327–37. doi: 10.1021/acsapm.1c01313 (PMC8762643; doi:10.1021/acsapm.1c01313)
Supplement: Supplementary file 1 — ap1c01313_si_001.pdf [file ap1c01313_si_001.pdf]

Supporting Information

Blending Linear and Cyclic Block Copolymers to Manipulate Nanolithographic Feature Dimensions

Authors: Amy D. Goodson,[1] Maxwell S. Rick[1], Jessie E. Troxler,[1] Henry S. Ashbaugh,[1*] and Julie N. L. Albert[1*]

[1]Department of Chemical and Biomolecular Engineering, Tulane University, New Orleans, Louisiana 70118, United States of America

Section 1: DPD Simulation Methods S-2

Section 2: Density Profiles S-5

References for Supporting Information..S-11

[*]Address correspondence to hanka@tulane.edu and jalbert6@tulane.edu.

Section 1: DPD Simulation Methods

Dissipative particle dynamics (DPD) is a coarse-grained simulation technique that represents the block copolymer (BCP) as a chain of soft beads, each representing 10s of monomers, connected by Hookean springs. The high degree of coarse graining and softness of the interactions permits DPD to examine the phase behavior and meso-scale structure of BCPs and polymer blends as a function of χ, N, f_A, and chain architecture.[1-5] Significantly, DPD retains the inherent thermal fluctuations eliminated from mean field theories that can be significant in polymer phase behavior.[1, 6-8]

Interparticle forces in DPD are broken up into a sum of pairwise conservative ($\boldsymbol{F}_{ij}^{C}$), dissipative ($\boldsymbol{F}_{ij}^{D}$), and random ($\boldsymbol{F}_{ij}^{R}$) forces between particles i and j. Interactions between bonded particles are modeled using a Hookean spring ($\boldsymbol{F}_{ij}^{S}$), which enforces bead connectivity and polymer architecture. The net force on DPD bead i is subsequently determined as a sum over interactions with all other beads in the simulation[14,15]

$$\boldsymbol{F}_i = \sum_{j\neq i} \boldsymbol{F}_{ij}^{C} + \boldsymbol{F}_{ij}^{D} + \boldsymbol{F}_{ij}^{R} + \boldsymbol{F}_{ij}^{S}. \tag{S1}$$

This force governs the time evolution of the particle system according to Newton's Laws of Motion; our simulations use the velocity Verlet algorithm[9] with a timestep $\Delta t = 0.025$. The mass of each bead in the simulation is assumed to be $m = 1$.

The conservative (energy-conserving) force enforces the chemical identity of the constituent coarse-grained bead, modeled in DPD as a soft repulsive interaction

$$\boldsymbol{F}_{ij}^{C} = \begin{cases} \frac{a_{ij}}{r_c}\left(1 - \frac{r_{ij}}{r_c}\right)\hat{\boldsymbol{r}}_{ij} & r_{ij} < r_c \\ 0 & r_{ij} \geq r_c \end{cases}, \tag{S2}$$

where a_{ij} is the DPD interaction parameter between beads i and j (dependent on the chemical identities of the interacting beads) that represents the maximum repulsion at complete overlap; r_{ij}

is the distance between interacting beads; $\hat{\boldsymbol{r}}_{ij} = \boldsymbol{r}_{ij}/r_{ij} = (\boldsymbol{r}_i - \boldsymbol{r}_j)/r_{ij}$ is the normalized direction vector pointing from j to i; and r_c is the cut-off distance after which inter-bead interactions vanish. For simplicity, we set $r_c = 1$ for all inter-bead interactions. The interaction between like beads, a_{AA} and $a_{\mathrm{BB},}$ which is set to 25, establishes the compressibility of the system while the interaction between unlike beads, a_{AB}, controls miscibility between the two blocks; for all simulations reported in this manuscript, a_{AB} was set to 65.[14] At a bead number density of $\rho = 3$ (the density of the simulations conducted here), the inter-bead interactions can be mapped to the Flory-Huggins χ parameter via the empirical correlation[1]

$$\chi = \frac{1}{3.27}(a_{\mathrm{AB}} - a_{\mathrm{AA}}). \tag{S3}$$

We note that since DPD is a coarse-grained simulation technique, we cannot directly compare simulation and experimental values of χ. Rather, DPD values of χ are typically calculated to match the experimental segregation strengths, i.e., $\chi N_{\mathrm{BCP}}|_{\mathrm{DPD}} = \chi N_{\mathrm{BCP}}|_{\mathrm{expt}}$, to affect a meaningful comparison between simulation and experiment.[5]

The dissipative force accounts for the viscous drag of the multiple atomic sites condensed onto a single coarse-grained bead. DPD models the dissipative force as

$$\boldsymbol{F}_{ij}^{D} = -\gamma\omega_{ij}^{D}(r_{ij})(\boldsymbol{v}_{ij}\cdot\boldsymbol{r}_{ij})\hat{\boldsymbol{r}}_{ij}, \tag{S4}$$

where γ is the friction coefficient, and $\boldsymbol{v}_{ij} = \boldsymbol{v}_i - \boldsymbol{v}_j$ is the relative velocity between particles i and j. The random force accounts for thermal Brownian kicks from the coarsened degrees of freedom, modeled in DPD as

$$\boldsymbol{F}_{ij}^{R} = -\sigma\omega_{ij}^{R}(r_{ij})\frac{\zeta_{ij}}{\sqrt{\Delta t}}\hat{\boldsymbol{r}}_{ij}, \tag{S5}$$

where σ is a constant noise amplitude related to the temperature, and ζ_{ij} is a Gaussian random number with a mean of zero and unit variance. The fluctuation dissipation theorem imposes the

following constraints on the distance dependent weight functions $\omega_{ij}^{D}(r)$ and $\omega_{ij}^{R}(r)$ and the amplitudes of the viscous and random forces[10]:

$$\omega_{ij}^{D}(r_{ij}) = \left[\omega_{ij}^{R}(r_{ij})\right]^{2} \tag{S6a}$$

and

$$\sigma^{2} = 2\gamma k_{B}T, \tag{S6b}$$

where $k_B T$ is the product of the Boltzmann's constant and the absolute temperature. Taken together then, eqs. (S4-S6) act as a thermostat ensuring DPD simulations sample the canonical (constant NVT) ensemble. Since the form of one of the weighting functions appearing in eq. (S4 and eq. (S5) is arbitrary, for simplicity DPD simulations adopt the expression

$$\omega_{ij}^{D}(r_{ij}) = \left[\omega_{ij}^{R}(r_{ij})\right]^{2} = \begin{cases} \left(1 - \frac{r_{ij}}{r_c}\right)^{2} & r_{ij} < r_c \\ 0 & r_{ij} \geq r_c \end{cases}, \tag{S7}$$

which vanishes beyond r_c, like the conservative force. Here, we assume $k_B T$ = 1 and σ = 3 (γ = 4.5) as recommended in ref. 6 to ensure fast, stable simulations.

Finally, the Hookean spring force in eq. (S1) is a second conservative interaction only between bonded beads that enforces intramolecular polymer connectivity. The spring force is evaluated as

$$F_{ij}^{S} = k r_{ij} \hat{\boldsymbol{r}}_{ij}, \tag{S8}$$

where k is the spring constant, assumed here to be equal to 4,[1, 7] independent of the chemical identity of the bonded monomers.

Section 2: Density Profiles

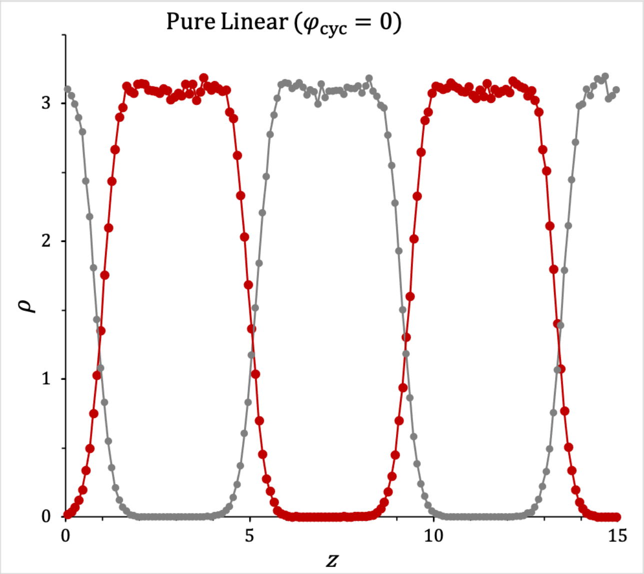

Figure S1. Concentrations of A (red circles and line) and B (gray circles and line) particles in a pure linear BCP with $N = 16$ along the direction normal to the lamellar interface.

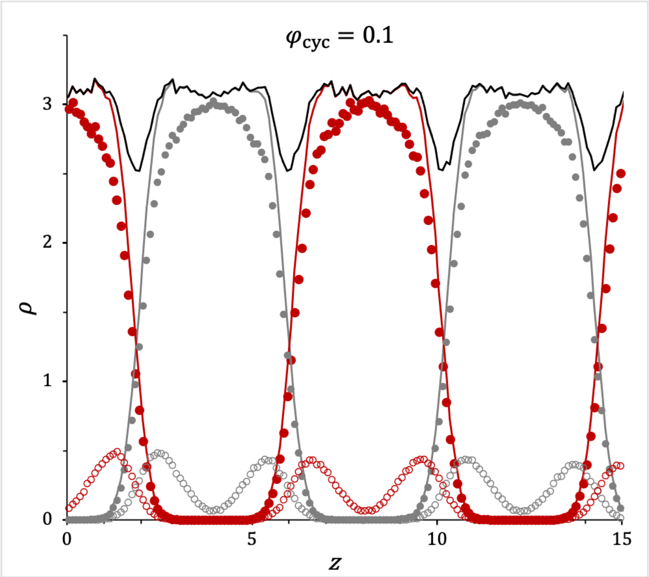

Figure S2. Concentrations of cyclic A (open red circles), cyclic B (open gray circles), linear A (solid red circles), and linear B (solid gray circles) in a cyclic/linear BCP blend with $\varphi_{\text{cyc}} = 0.1$ and $N = 16$ along the direction normal to the lamellar interface. Lines represent total block A (red line), block B (gray line), and overall (black line) monomer concentrations.

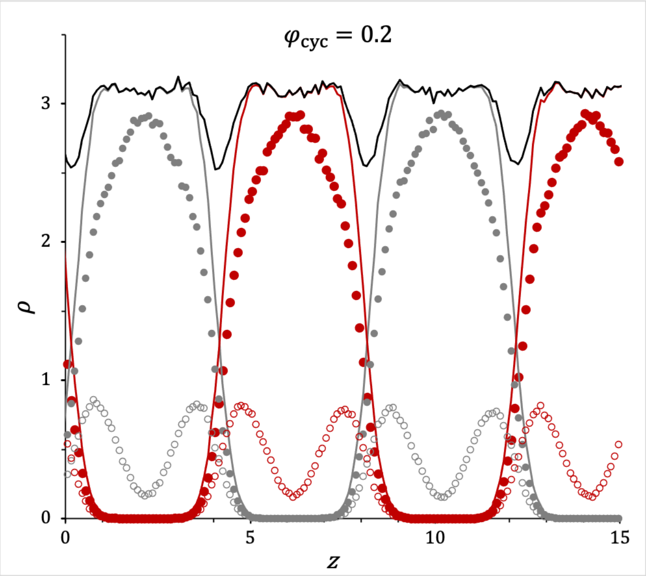

Figure S3. Concentrations of cyclic A (open red circles), cyclic B (open gray circles), linear A (solid red circles), and linear B (solid gray circles) in a cyclic/linear BCP blend with $\varphi_{\mathrm{cyc}} = 0.2$ and $N = 16$ along the direction normal to the lamellar interface. Lines represent total block A (red line), block B (gray line), and overall (black line) monomer concentrations.

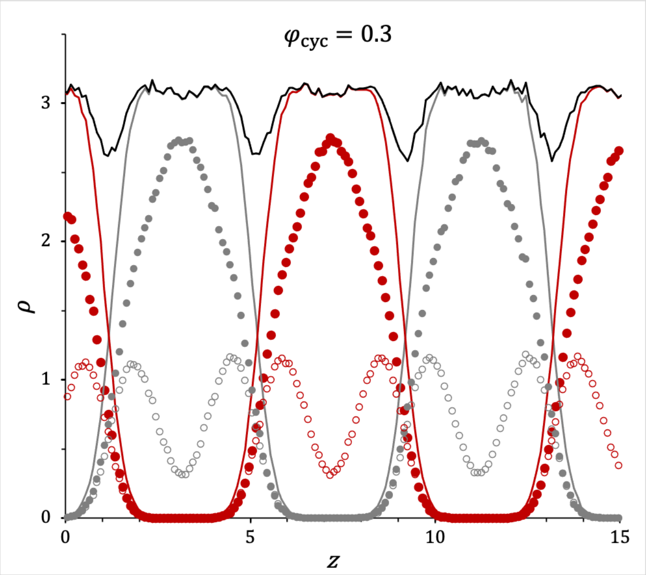

Figure S4. Concentrations of cyclic A (open red circles), cyclic B (open gray circles), linear A (solid red circles), and linear B (solid gray circles) in a cyclic/linear BCP blend with $\varphi_{\mathrm{cyc}} = 0.3$ and $N = 16$ along the direction normal to the lamellar interface. Lines represent total block A (red line), block B (gray line), and overall (black line) monomer concentrations.

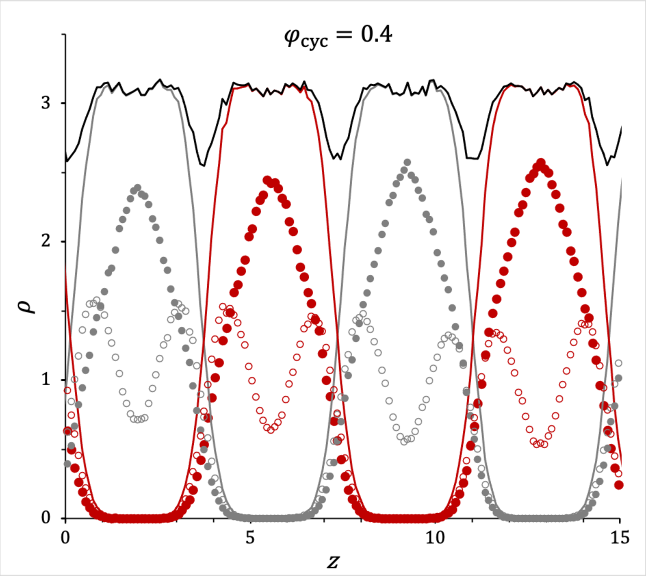

Figure S5. Concentrations of cyclic A (open red circles), cyclic B (open gray circles), linear A (solid red circles), and linear B (solid gray circles) in a cyclic/linear BCP blend with $\varphi_{\mathrm{cyc}} = 0.4$ and $N = 16$ along the direction normal to the lamellar interface. Lines represent total block A (red line), block B (gray line), and overall (black line) monomer concentrations.

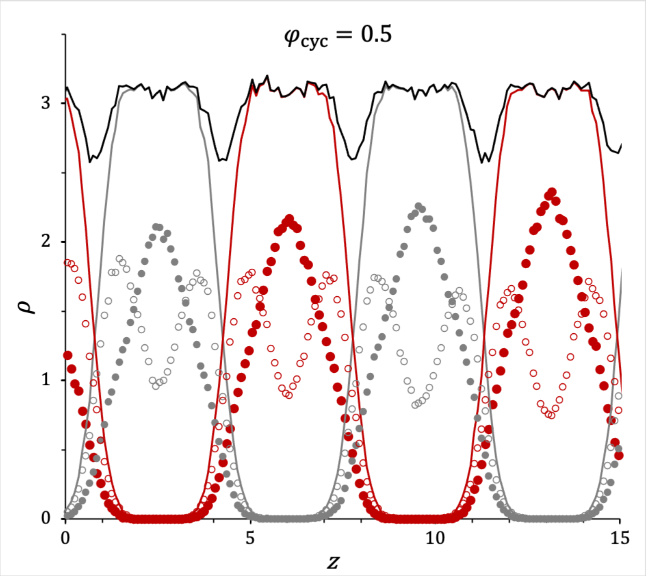

Figure S6. Concentrations of cyclic A (open red circles), cyclic B (open gray circles), linear A (solid red circles), and linear B (solid gray circles) in a cyclic/linear BCP blend with $\varphi_{\mathrm{cyc}} = 0.5$ and $N = 16$ along the direction normal to the lamellar interface. Lines represent total block A (red line), block B (gray line), and overall (black line) monomer concentrations.

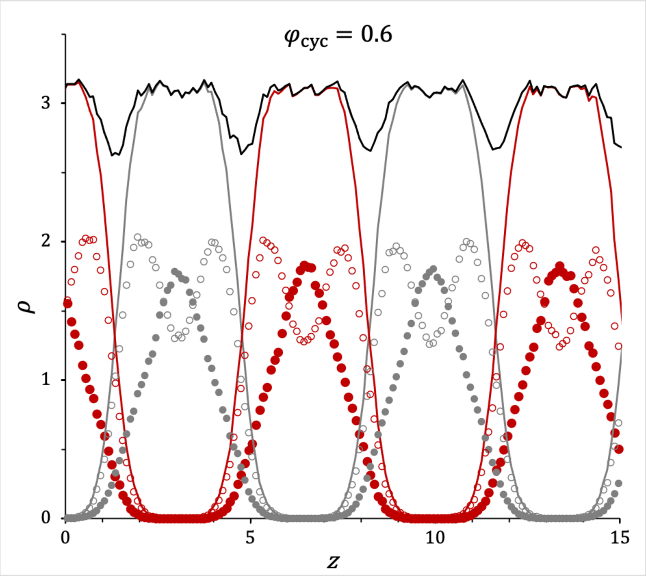

Figure S7. Concentrations of cyclic A (open red circles), cyclic B (open gray circles), linear A (solid red circles), and linear B (solid gray circles) in a cyclic/linear BCP blend with $\varphi_{\text{cyc}} = 0.6$ and $N = 16$ along the direction normal to the lamellar interface. Lines represent total block A (red line), block B (gray line), and overall (black line) monomer concentrations.

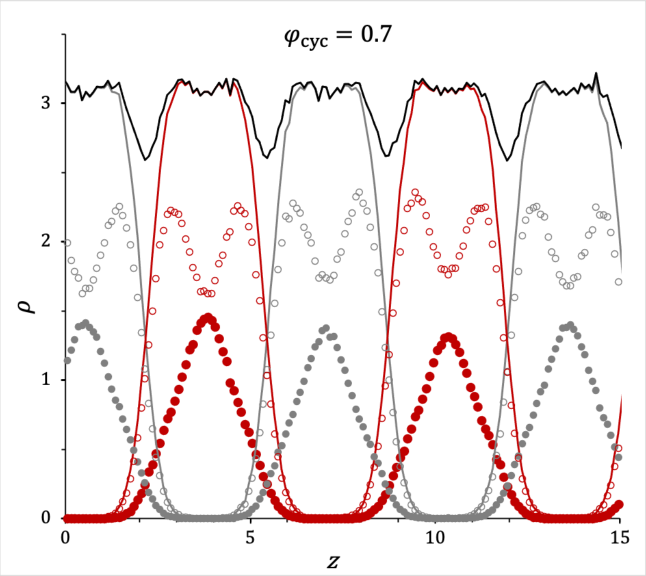

Figure S8. Concentrations of cyclic A (open red circles), cyclic B (open gray circles), linear A (solid red circles), and linear B (solid gray circles) in a cyclic/linear BCP blend with $\varphi_{\text{cyc}} = 0.7$ and $N = 16$ along the direction normal to the lamellar interface. Lines represent total block A (red line), block B (gray line), and overall (black line) monomer concentrations.

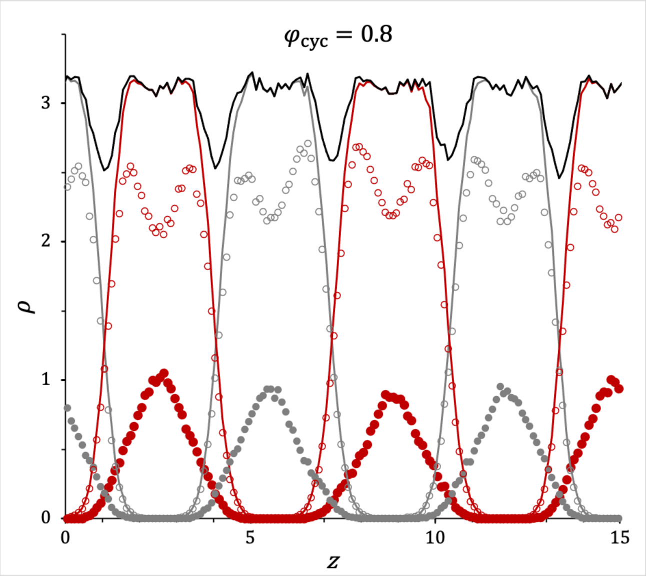

Figure S9. Concentrations of cyclic A (open red circles), cyclic B (open gray circles), linear A (solid red circles), and linear B (solid gray circles) in a cyclic/linear BCP blend with $\varphi_{cyc} = 0.8$ and $N = 16$ along the direction normal to the lamellar interface. Lines represent total block A (red line), block B (gray line), and overall (black line) monomer concentrations.

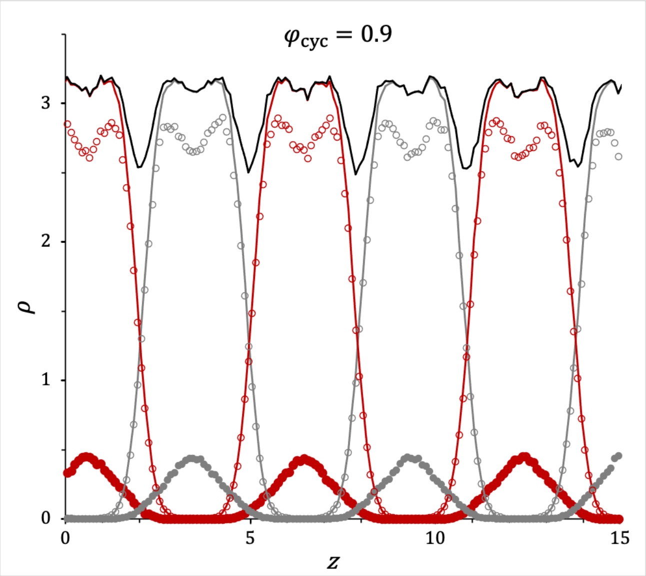

Figure S10. Concentrations of cyclic A (open red circles), cyclic B (open gray circles), linear A (solid red circles), and linear B (solid gray circles) in a cyclic/linear BCP blend with $\varphi_{cyc} = 0.9$ and $N = 16$ along the direction normal to the lamellar interface. Lines represent total block A (red line), block B (gray line), and overall (black line) monomer concentrations.

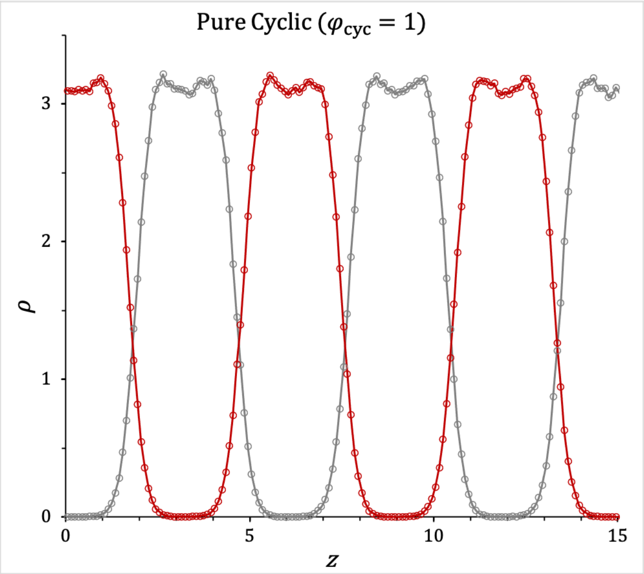

Figure S11. Concentrations of A (red circles and line) and B (gray circles and line) particles in a pure cyclic BCP with $N = 16$ along the direction normal to the lamellar interface.

References

1. Groot, R. D.; Madden, T. J. Dynamic simulation of diblock copolymer microphase separation. *The Journal of Chemical Physics* **1998,** 108 (20), 8713-8724 DOI: 10.1063/1.476300.

2. Qian, H.-J.; Lu, Z.-Y.; Chen, L.-J.; Li, Z.-S.; Sun, C.-C. Computer Simulation of Cyclic Block Copolymer Microphase Separation. *Macromolecules* **2005,** 38 (4), 1395-1401 DOI: 10.1021/ma0478658.

3. Gavrilov, A. A.; Kudryavtsev, Y. V.; Chertovich, A. V. Phase diagrams of block copolymer melts by dissipative particle dynamics simulations. *The Journal of chemical physics* **2013,** 139 (22), 224901.

4. Posel, Z.; Lísal, M.; Brennan, J. K. Interplay between microscopic and macroscopic phase separations in ternary polymer melts: Insight from mesoscale modelling. *Fluid Phase Equilibria* **2009,** 283 (1), 38-48 DOI: https://doi.org/10.1016/j.fluid.2009.05.014.

5. Soto-Figueroa, C.; Rodríguez-Hidalgo, M.-d.-R.; Martínez-Magadán, J.-M.; Vicente, L. Dissipative Particle Dynamics Study of Order−Order Phase Transition of BCC, HPC, OBDD, and LAM Structures of the Poly(styrene)−Poly(isoprene) Diblock Copolymer. *Macromolecules* **2008,** 41 (9), 3297-3304 DOI: 10.1021/ma7028264.

6. Groot, R. D.; Warren, P. B. Dissipative particle dynamics: Bridging the gap between atomistic and mesoscopic simulation. *The Journal of Chemical Physics* **1997,** 107 (11), 4423-4435 DOI: 10.1063/1.474784.

7. Groot, R. D.; Madden, T. J.; Tildesley, D. J. On the role of hydrodynamic interactions in block copolymer microphase separation. *The Journal of Chemical Physics* **1999,** 110 (19), 9739-9749 DOI: 10.1063/1.478939.

8. Nikunen, P.; Vattulainen, I.; Karttunen, M. Reptational dynamics in dissipative particle dynamics simulations of polymer melts. *Physical Review E* **2007,** 75 (3), 036713.

9. Allen, M. P.; Tildesley, D. J., *Computer simulation in chemical physics*. Springer Science & Business Media: 2012; Vol. 397.

10. Espanol, P.; Warren, P. Statistical mechanics of dissipative particle dynamics. *EPL (Europhysics Letters)* **1995,** 30 (4), 191.
